# Vertebral Compression Fractures after Lumbar Instrumentation

**DOI:** 10.7759/cureus.1729

**Published:** 2017-09-29

**Authors:** Michelle Granville, Aldo Berti, Robert E Jacobson

**Affiliations:** 1 Miami Neurosurgical Center, University of Miami Hospital

**Keywords:** lumbar stenosis, degenerative spondylolisthesis, lumbar spinal instrumentation, osteoporotic vertebral compression fractures, adjacent level vertebral fractures, sacral insufficiency fractures

## Abstract

Lumbar spinal stenosis (LSS) is primarily found in an older population. This is a similar demographic group that develops both osteoporosis and vertebral compression fractures (VCF). This report reviewed a series of patients treated for VCF that had previous lumbar surgery for symptomatic spinal stenosis. Patients that only underwent laminectomy or fusion without instrumentation had a similar distribution of VCF as the non-surgical population in the mid-thoracic, or lower thoracic and upper lumbar spine. However, in the patients that had previous short-segment spinal instrumentation, fractures were found to be located more commonly in the mid-lumbar spine or sacrum adjacent to or within one or two spinal segments of the spinal instrumentation. Adjacent-level fractures that occur due to vertebral osteoporosis after long spinal segment instrumentation has been discussed in the literature. The purpose of this report is to highlight the previously unreported finding of frequent lumbar and sacral osteoporotic fractures in post-lumbar instrumentation surgery patients. Important additional factors found were lack of preventative medical treatment for osteoporosis, and secondary effects related to inactivity, especially during the first year after surgery.

## Introduction

**Figure 1:**
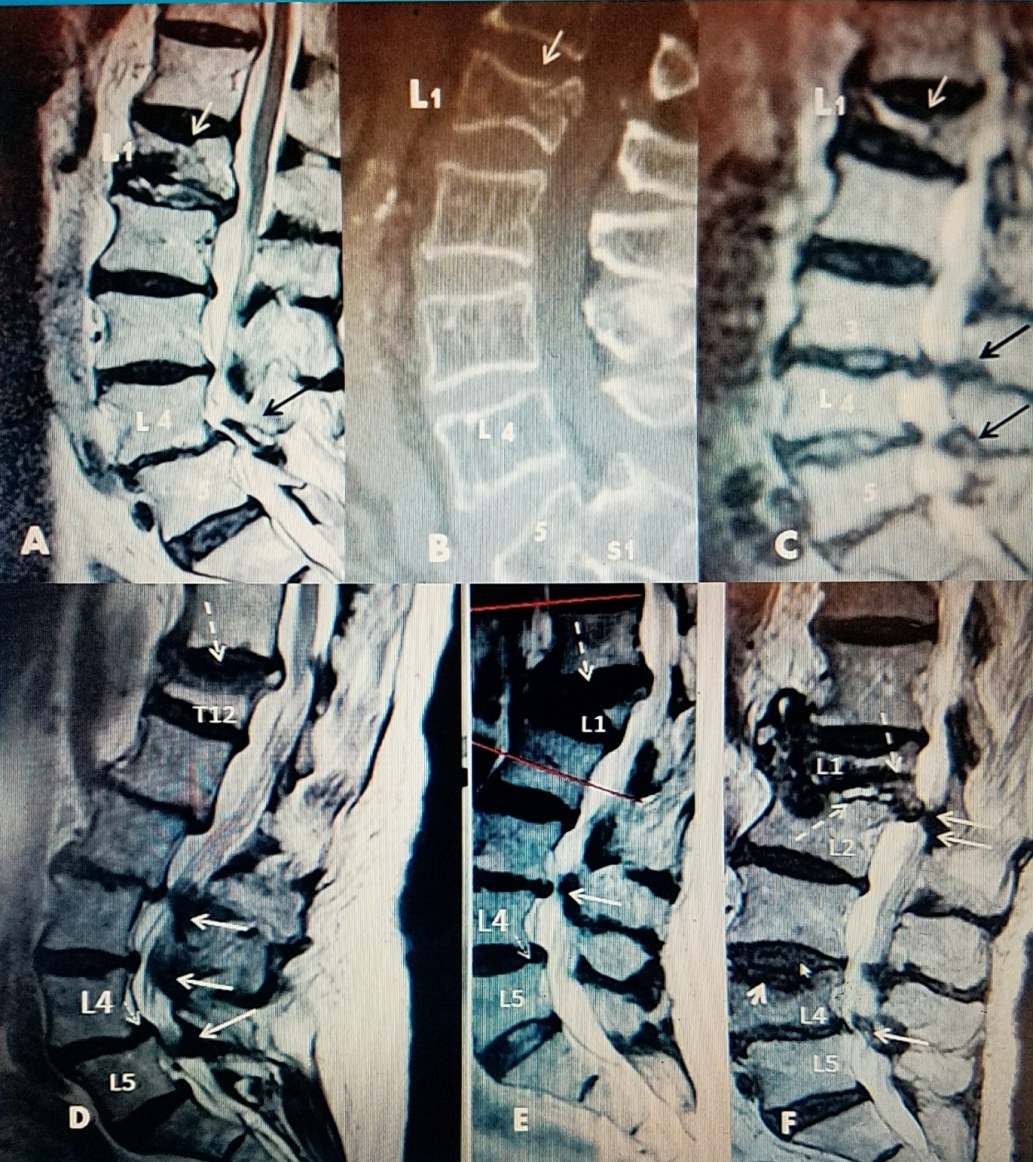
Examples of thoracic and upper lumbar fracture (fx) with lumbar spinal stenosis A: Sagittal T2 magnetic resonance imaging (MRI) scan in a 78-year-old female with preexisting L4-5 spondylolisthesis (black arrow), and new vertebral compression fracture (VCF) at L1 (white arrow) B: Sagittal computerized tomography (CT) scan of a 74-year-old female with grade 2 L5-S1 spondylolisthesis and acute L1 VCF (white arrow) C: Sagittal T2 MRI scan of a 85-year-old male with asymptomatic stenosis L3-4, L4-5 and spondylosis with acute L1 VCF (white arrow) evolving into a Vertebrae Plana D: Sagittal T2 MRI of a 72-year-old male with acute T12 70% wedge compression fracture at T12 (dotted white arrow), L2 retrolisthesis with L2-3, L3-4 and L4-5 spondylosis, and stenosis with grade 1 spondylolisthesis at L4-5 (small solid white arrow) E: Sagittal T2 MRI of a 76-year-old female with acute L1 compression fracture with a 90% collapse with kyphotic angulation (dotted white arrow). There is grade 1 anterolisthesis at L4-5 (thin white arrow). There is L3-4 spondylosis with sagittal stenosis (solid white arrow). F: Sagittal T2 MRI of a 71-year-old female with post kyphoplasty collapse at L1 (dotted white arrow) with posterior canal compression (2 white arrows) and early edema of anterior superior endplate of L2. A new edematous fracture of superior endplate of L4 (small solid white arrow) with posterior stenosis at L4-5 (single solid white arrow).

As the population becomes older, it is common to see patients developing concurrent spinal problems such as lumbar spinal stenosis (LSS), degenerative spondylolisthesis (DSL), and osteoporotic vertebral compression fractures (VCF). These conditions often affect the same or adjacent regions of the thoracic and lumbar spine [1-3]. Large radiologic and anatomic studies have shown a biphasic distribution of VCF with 45% to 55% found in the mid-thoracic spine from T6 to T8, and 10% to 20% from T12 to L2 [4]. Lumbar and sacral fractures make up a small percentage of VCF. Magnetic resonance imaging (MRI) and computerized tomography (CT) scans are a key part of the diagnosis. Often patients are being studied for one problem, such as a traumatic VCF, and are found to have previously asymptomatic lumbar stenosis or spondylolisthesis; or the reverse, where a patient is being evaluated for radicular pain or neurogenic claudication, and is found to have a previous VCF [3, 5] (Figure 1).

Further evidence of the interconnection is highlighted in studies finding that there is increased risk of falls, and consequently risk of VCF, in patients with LSS [6]. Less activity in patients with mild to moderately symptomatic lumbar stenosis can lead to progression of underlying osteoporosis with a corresponding higher risk of fractures if they fall because of poor gait, secondary to stiffness as well as motor and sensory deficits from the lumbar stenosis [1, 6]. Frequently, patients with lumbar stenosis, concomitant facet degeneration, and hypertrophy have altered spinal mechanics with increased spinal rigidity, worsening gait, and abnormal balance; all creating a higher risks for falls [7].

Surgical treatment for lumbar stenosis can also increase the risk of worsening osteoporosis leading to subsequent fractures after surgery. Both LSS and degenerative spondylolisthesis are often treated with multilevel lumbar laminectomy, combined with the use of segmental fixation with pedicle or cortical screws. Untreated or progressive osteoporosis can lead to an increased risk of screw loosening, screw pullout, fractures at the level of the superior screw, and adjacent level fractures [8]. Prolonged rehabilitation can further aggravate underlying osteoporosis. The post-surgical fracture risk in patients undergoing longer multilevel lumbar or lumbo-thoracic instrumentation has been found to be significantly higher in the initial six to 12 months after surgery because of immobility, often leading to increased postsurgical osteoporosis in already elderly patients [9].

## Materials and methods

A retrospective review of charts, MRI and CT scans of patients with single or multilevel, acute or sub-acute osteoporotic fractures that also had previous lumbar surgery for lumbar stenosis was performed. These patients were evaluated in the office of the neurological surgery practice of the authors over a 24-month time period extending from January 2015 through January 2017. Relevant clinical history including levels and type of fusion and instrumentation, time interval between surgery and fracture, previous treatment for osteoporosis, comorbidities, sex, and age of the patients were recorded. Other radiologic findings were noted as well as evidence of joint replacement in the hip or knee. This group was compared to patients without previous lumbar surgery regarding type, number, and level of fractures.

## Results

There were a total of 10 patients identified that had previous surgery for lumbar stenosis and developed a subsequent VCF. Seven patients had been followed after stenosis surgery within the previous five years, having been followed since their original surgery. The other three patients had surgery at other centers, but were subsequently evaluated for a new VCF three to 11 years after their previous lumbar stenosis surgery. There were 12 fractures in the 10 patients, with two patients presenting with multiple fractures. There were seven female patients and three male patients. The average patient age was 77.4 years, with the youngest being 71 years old and the oldest 82. There were six thoracic fractures, four lumbar fractures, and two sacral fractures. Four of the six thoracic fractures were at T12. There were two lumbar fractures located at L3 and one each located at L2 and L5. There were two sacral fractures; one associated with an L5 fracture. Range of time from instrumentation to development of a new fracture was less than six months to eight years. Six of 10 patients developed a fracture within six months to one year of lumbar surgery. Although the patient group with previous lumbar surgery for stenosis was small, there were a much higher number of low lumbar and sacral fractures seen in this group compared to the normal distribution of VCF in the mid and lower thoracic and upper lumbar distribution for osteoporotic VCF’s. Thoracic fractures seen in this group were also concentrated at T12 with only 1 fracture at T9; none had fractures at higher levels. Interestingly, two patients with posterior interlaminar stabilization devices had fractures, one at the adjacent level, many years after the lumbar surgery for stenosis (Table 1). 

**Table 1:** Table showing patient age, sex, surgery performed, level of fracture, time between surgery and fracture, and if the patient had a previous fracture. NA=Not Available; * Procedure after fracture; MO=Months YR=Year; SX=Symptoms

AGE (years)	SEX	SURGERY PERFORMED	FRACTURE	# OF YEARS	FRACTURE BEFORE SX
78	F	L4-5 PEDICLE SCREWS W/INSTRUMENTATION	L2	11 YRS	NO
74	F	L 2-3 X-STOPR interspinous device, L3-4 PEDICLE SCREWS W/INSTRUMENTATION	T12	6 YRS	NO
71	F	L4-5 X-STOPR interspinous device	L3	9 YRS	NO
82	M	L3-L5 PEDICLE SCREWS W/INSTRUMENTATION	SACRUM	1 YR	NO
78	F	T11-12 & L2-3 CEMENTED PEDICLE SCREW INSTRUMENTATION	L5 & SACRUM	< 1 YR	YES
81	F	L4-5 AND L5-S1 INSTRUMENTATION	T10 & T12	< 1 YR	YES
79	M	L3-4 AND L4-5 LAMINECTOMIES	T9	<1 YR	NO
81	F	LAMINECTOMY IN THE PAST & SPONDYLOLISTHESIS	T12	6 YRS	NO
74	M	L3, L4, L5 & S1 LAMINECTOMIES	L3	< 1 YR	NO
76	F	L4-5 & L5-S1 LAMINECTOMIES	T12	*> 6 MO	YES

## Discussion

Lumbar degenerative disease and LSS is more common over the age of 55. This same demographic group, especially females, is more prone to developing osteoporosis, and is at risk for developing osteoporotic VCF. Clinically, lumbar stenosis can vary from being asymptomatic to causing neurogenic claudication. Between 40% to 70% of patients with radiographic stenosis on MRI scan are asymptomatic, so lumbar stenosis can be frequently seen concurrently in an MRI scan performed after a fall for possible VCF [10]. MRI scans in patients with stenosis can demonstrate a range from extremely localized stenosis, especially if associated with degenerative spondylolisthesis, to diffuse multilevel degenerative changes and canal narrowing [11]. Studies have shown that patients with LSS have decreased physical activity and associated depression which leads to actual measurable metabolic changes that worsen their underlying osteoporosis [1-2].

**Figure 2:**
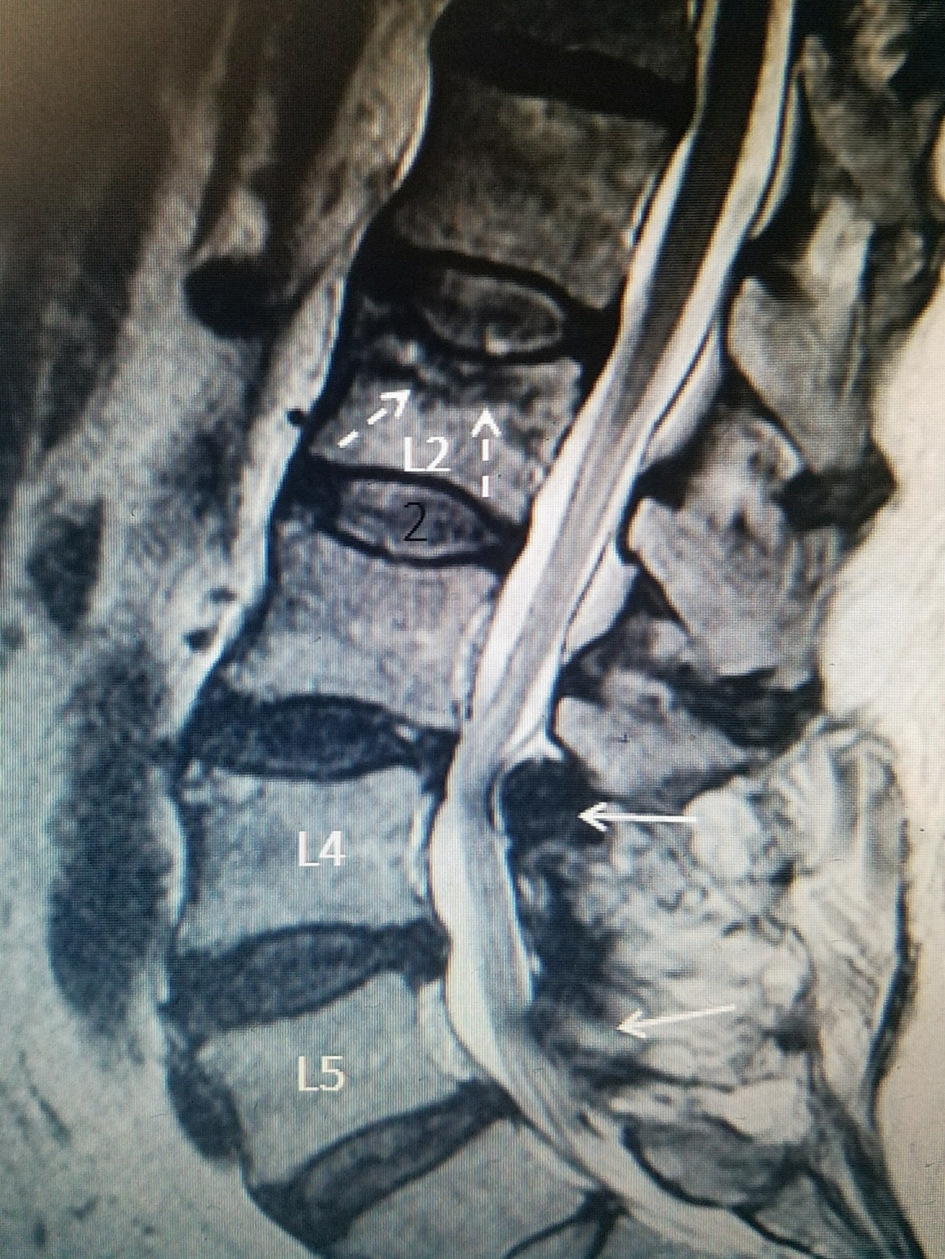
L2 fracture 15 years after lumbar fusion above post fusion stenosis after previous L4 to S1 fusion Sagittal T2 magnetic resonance imaging (MRI) scan: Patient is a 68-year-old female with previous L4-S1 laminectomy and posterior fusion 15 years previously without instrumentation. There is development of post fusion stenosis at L3-4 (solid white arrows). After a fall, she developed mid lumbar pain. MRI revealed an acute superior endplate compression fracture at L2 (dotted white arrows).

Once patients with stenosis are symptomatic and develop chronic pain and neurologic symptoms, they often undergo surgery, although the type and extent of surgery may vary. Surgery often includes lumbar decompression with or without instrumentation. If there is instability, instrumentation may include rigid spinal fusion systems such as posterior lumbar interbody fusions (PLIF), transforaminal interbody fusion (TLIF), fixation with pedicle screws (PSF), cortical screws (CS) with both short segment and longer multi-segment constructs, and interspinous decompression systems [11]. Early experience with spinal instrumentation for lumbar stenosis or spondylolisthesis reported up to a 28% incidence of later development of adjacent level fractures, again more common in postmenopausal females. Adjacent level fractures are found at, or adjacent to, the upper level of instrumentation, particularly when there is greater than two or more levels of instrumentation [9]. It was found that the subsequent fractures were not specifically related to the type of instrumentation used. However, patients undergoing longer multilevel instrumentation clearly had a higher frequency of fractures [9, 12]. Patients can also develop either a unilateral or bilateral sacral insufficiency fracture if the lower lumbosacral area is not included in the original fusion with instrumentation [13-14]. Osteoporotic fractures have also been reported within the instrumented construct as well as the pedicle where screws were placed. Underlying severe osteoporosis is considered a significant factor in these type of cases where the vertebral fracture is within the boundaries of the instrumentation [15-18]. Osteoporosis can directly affect the hold of the screws placed during surgery, especially when superimposed on pre-existing osteopenia and osteoporosis. This can lead to loosening of the screws, localized pain, screw pullout, and fractures at the interface between instrumented and non-instrumented levels [19]. The relative inactivity of the patients post spinal instrumentation has been documented to lead to increased loss of bone density. This is loss of bone density is more marked within the first year after surgery [2, 9, 20]. Patients can also develop iatrogenic stenosis many years after a lumbar fusion, leading again to spinal rigidity and immobility. The immobility of the fused stenotic spinal segment, with or without instrumentation, leads to restriction of lower lumbar motion, and a higher incidence of falls that can lead to VCF [8]. This appears to have a greater impact on the upper level of the fusion, and when combined with later development of osteoporosis, can lead to fractures close to the spinal fusion [19-20] (Figure 2).

**Figure 3:**
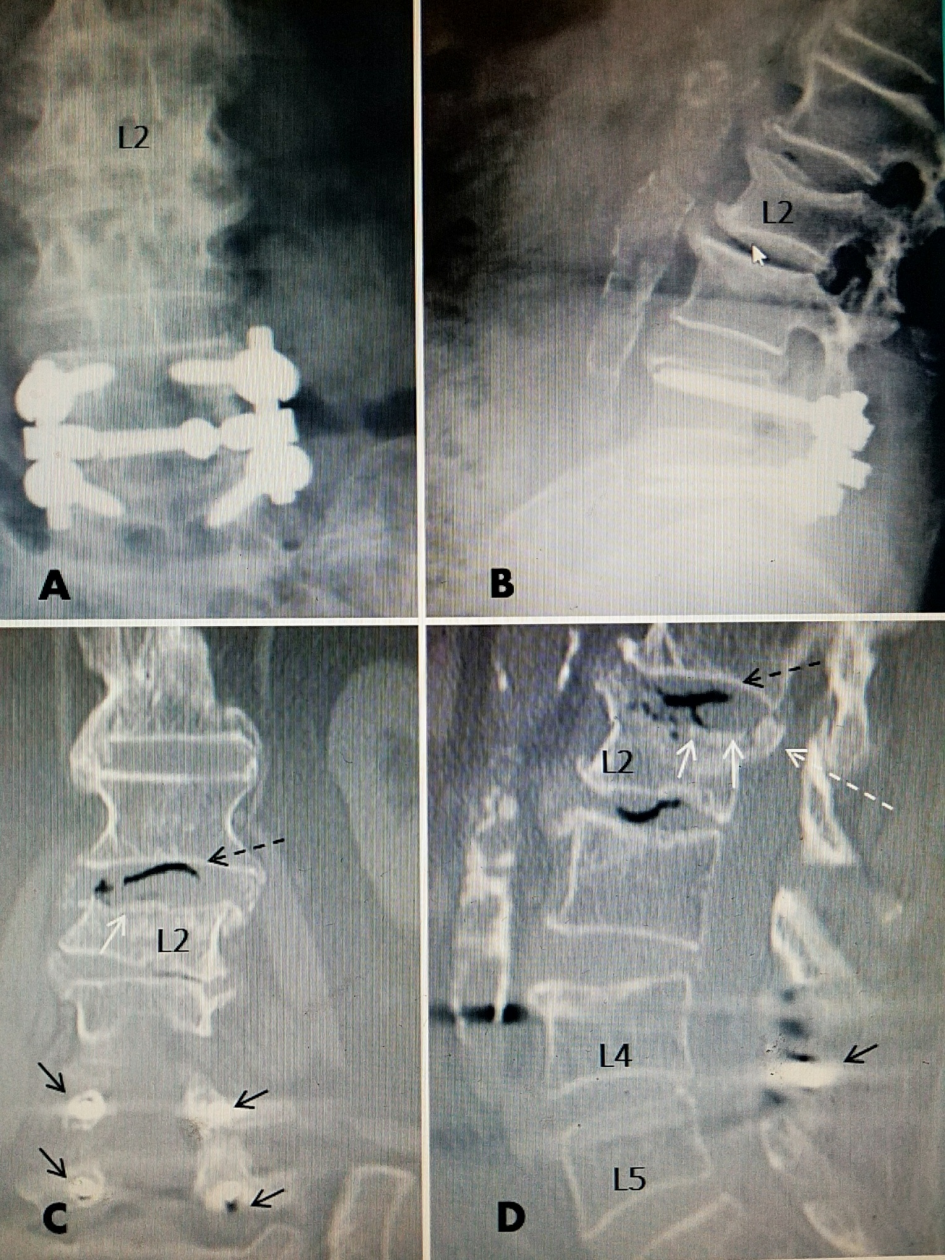
L2 compression fracture after fall above previous L4-5 pedicle fixation A, B: Anterior-posterior (AP) and lateral films showing rigid L4-5 fixation and superior endplate fracture L2. C: Coronal (C) reconstructed computerized tomography (CT) scan showing in greater detail the collapsed fracture of the superior endplate. There is a black signal in disc space at L1-2 (dotted black arrow). The L4 and L5 pedicle screws can be seen in proper position (solid small black arrows) D: Sagittal reconstructed CT. There is fracture and posterior displacement of the superior endplate into the spinal canal (dotted white arrow). Comminuted fracture of superior endplate of L2 (dotted white arrows). There is necrotic aseptic vacuum change at L1-L2 disc and also at L2-3 disc shown by black signal on CT scan (dotted black arrow). The cross link connecting the two lateral rods is seen between the spinous process at L4-5 (small black arrow).

Previous reports had focused on fractures at the upper level or adjacent level to multilevel instrumentation [9-10, 12]. Of the 10 patients reviewed in this series, four patients developed VCF after a decompressive laminectomy without fixation. In this small group, three of the four patients had fractures in the lower thoracic spine between T9 and T12. This is similar to patients without surgery, and large studies of patients with VCF [4]. However, in our small group, the one patient that had an L2 fracture had a posterolateral lumbar fusion, suggesting that even without instrumentation, loss of motion may increase the risk of adjacent level lumbar fractures. In our review, six patients had spinal instrumentation and developed osteoporotic VCF at some time after surgery. Four of the six patients had spinal fixation with pedicle or cortical screws, and two had one-level posterior interspinous distraction and stabilization devices. In the patients with fractures after pedicle fixation, only one patient had longer multilevel instrumentation, and the other three had one or two-level instrumentation at L4-5 and L5-S1. Fractures were identified at the level of the upper screw or one level above the instrumentation in the lumbar spine in two patients, and at the sacrum in another two patients. Two of the four patients also had multiple fractures (Figure 3).

**Figure 4:**
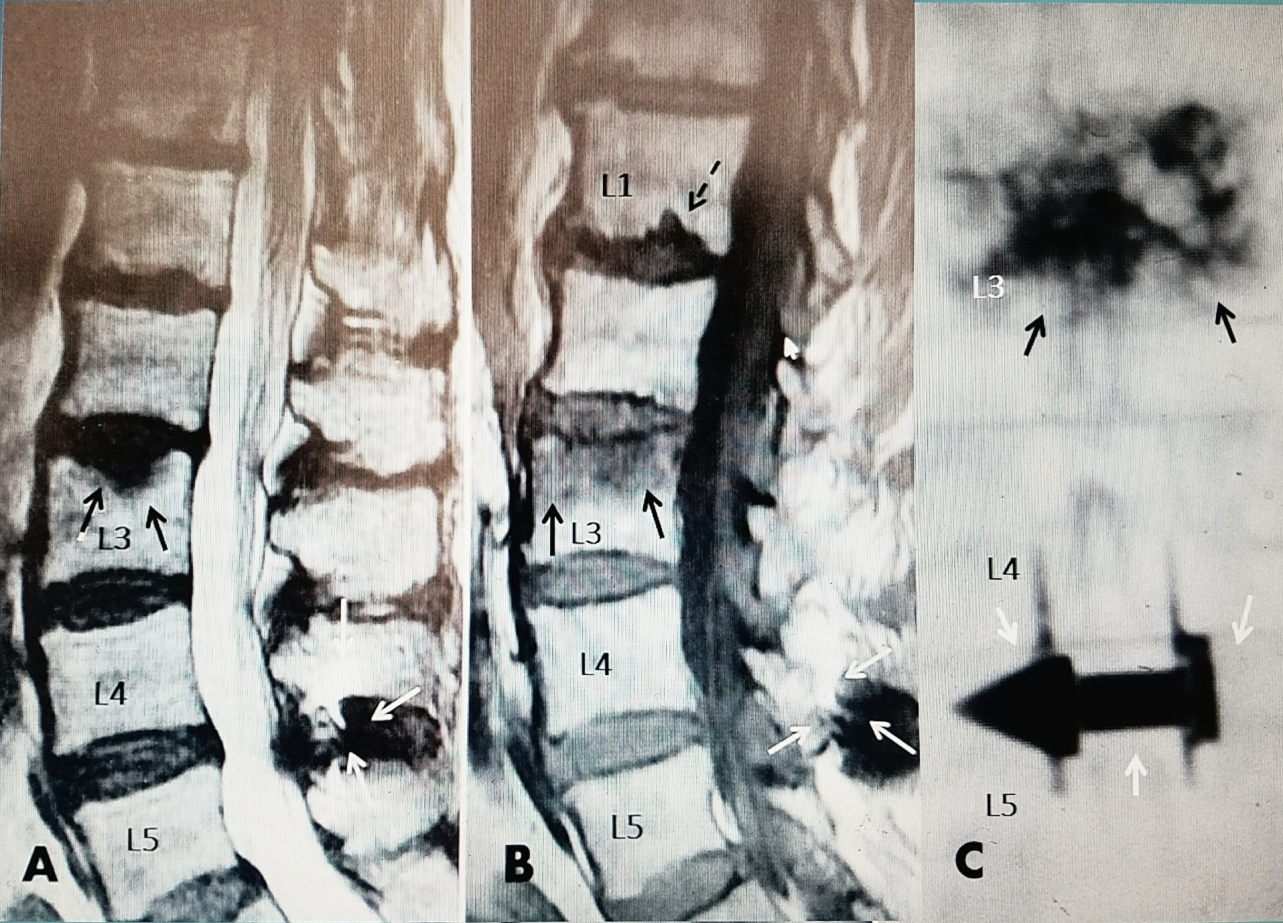
L3 osteoporotic compression fracture five years after L4-5 interspinous distraction device A: Sagittal T2 (MRI) showing acute L3 superior endplate compression fracture (solid black arrows) above L4-5 interspinous distraction device (solid white arrows). B: T1 sagittal MRI showing 50% edema in superior L3 vertebrae (solid black arrows). There is a Schmorl's node or possible second fracture at the inferior endplate of L1 (dotted black arrow). Interspinous device solid white arrows at L4-5. C: Postoperative AP X-rays showing bilateral vertebroplasty cement at L3 (black arrows) and L4-5 interspinous distraction device (white arrows).

Patients that undergo lumbar surgery for either degenerative spondylolisthesis or single-level stenosis may have single-level posterior instrumentation with an interspinous distraction device instead of pedicle fixation [5, 21]. There were two patients in our review that had interspinous stabilization and distraction devices inserted. In both of these patients, the lumbar fractures were located one and two segments above the interspinous device. Interestingly, even though the fixation device is placed posteriorly in the interspinous and intralaminar space and not inserted directly into the osteoporotic vertebral bodies, these patients are also at risk of getting adjacent level fractures (Figure 4).

There are multiple factors affecting the timing, type, and location of fractures seen after lumbar instrumentation for stenosis. With the growing use of instrumentation in an older osteoporotic population, there are increasing reports of the development of adjacent-level fractures above spinal instrumentation, especially when there are two or more levels of instrumentation [9, 12]. The use of cortical screws rather than pedicle screws has partially decreased the frequency of fractures at the level of screw insertion. Using unicortical screws that do not violate the anterior cortex has less risk of upper segment fracture than bicortical screws. Despite this, up to 10% of patients, even with unicortical screws, need further remedial surgery after instrumentation and fusion, due to this upper segmental failure at the bone-screw interface [9]. 

The risk of development of adjacent-level segmental degeneration and fractures at the uppermost level of fixation with either pedicle or cortical screws is well recognized [8-9, 12, 20]. However, as we found in this series, it can even occur after use of one-level interspinous stabilization device [21]. These devices are gaining wider use, so it is important to note the possible development of either adjacent level fractures, or upper lumbar and lower thoracic fractures, as in the two cases in this series. In the literature and our patient group, osteoporotic vertebral fractures are found to occur most frequently in the first six to 24 months after instrumentation, and often are clinically indicated by the onset of new pain or delayed onset of pain and deformity that is often detected after a minor fall [22-23]. The development of vertebral compression fractures after lumbar spinal instrumentation is influenced by patient age, pre-existing osteoporosis, number of levels fused, and whether the sacrum was included in the construct [24-25]. Postoperative inactivity leads to documented increased loss of bone density, and makes these patients' risk for fracture after surgery even higher [2, 20]. All of our cases that developed subsequent fractures also had untreated osteopenia or osteoporosis before the fracture was detected. The fact that previous one or two-level lumbar spinal instrumentation can affect the location of subsequent osteoporotic vertebral fractures has not been recognized. This report highlights that even patients undergoing single or double-level instrumentation are at risk for these fractures.

## Conclusions

In this study, patients with pre-existing symptomatic LSS that had spinal surgery with spinal instrumentation had a higher percentage of lumbar or sacral fractures than either patients with lumbar surgery without instrumentation or with asymptomatic lumbar stenosis. Upper segment and adjacent segment fractures have been related previously to long spinal segment instrumentation. This report highlights that osteoporotic lumbar fractures can also occur with either one or two-level pedicle instrumentation. It is also important to note that these fractures can occur in patients with previous insertion of posterior interspinous fixation devices. Fractures in these cases were found within one or two vertebral segments of the spinal instrumentation. One or two-level instrumentation in combination with osteoporosis increased the risk for development of VCF fractures within the first 12 months after surgery. Often these patients are braced and restricted from strong activity for three to six months after surgery. Studies have documented definite decreased bone mineral density due to lack of exercise after spinal instrumentation. Important underlying factors for risk of developing postoperative VCF are the presence of untreated osteoporosis (often worsened due to relative inactivity secondary to the original symptomatic lumbar stenosis) and subsequent surgery. Better postsurgical activity and physical therapy combined with aggressive medical treatment of underlying osteoporosis are important in preventing subsequent fractures in these patients.
